# Who will treat older patients? Should medical education focus more on activities aimed at displaying positive attitudes toward older people? The prevalence of ageism among students of medical and health sciences

**DOI:** 10.3389/fpubh.2022.1032487

**Published:** 2022-12-01

**Authors:** Marta Podhorecka, Jakub Husejko, Agnieszka Woźniewicz, Anna Pyszora, Kornelia Kȩdziora-Kornatowska

**Affiliations:** ^1^Department of Geriatrics, Collegium Medicum in Bydgoszcz, Nicolaus Copernicus University in Toruń, Bydgoszcz, Poland; ^2^Department of Palliative Care, Collegium Medicum in Bydgoszcz, Nicolaus Copernicus University in Toruń, Bydgoszcz, Poland

**Keywords:** ageism, gerontology, attitudes toward the elderly, education system, public health

## Abstract

In the conditions of an aging society, a justification is found to explain the issue of the attitude of future health care workers, who are to care for elderly people in the future, toward these people, which will make it possible to predict in advance what problems related to the care of seniors may soon arise. After clarifying this issue, it will be important to distinguish the individual factors influencing this attitude in order to determine which social phenomena will require special attention. Eight hundred and three medical and health sciences students participated in the research from various fields of medical studies. A Survgo system was used, where an online questionnaire was placed and then posted in social media. Only students of medicine and healthcare facilities participated in the study. The first questionnaire contained socio-demographic questions. Then, the KOAP questionnaire and Welch's *t*-test were used, and finally the proprietary questionnaire on contact with seniors. Analyses were conducted using the R Statistical language. Scores on the KAOP questionnaire ranged from 122.4 to 134.57. The highest scores were shown for physiotherapy students and the lowest for pharmacy students. The highest level of attitude toward elderly was reported in students of 1st to 3rd year of study. For 4th-5th year or supplementary level students, attitude levels were decreasing. Welch's *t*-test showed that the level of attitude toward old people in men was significantly higher than in women. There was no significant correlation between the age factor on the quotient scale and the KOAP score. Married individuals had significantly higher KAOP scores compared to singles and those in an informal relationship. No significant differences between the study stage factor and KAOP score have been found. Those who live and/or have regular contact with the elderly were characterized by higher KAOP questionnaire scores. The attitude toward the elderly depends on many factors, such as the chosen field of study, stage of education, contact with the elderly, gender or marital status. In order to develop proper contact with seniors, the solution may be to influence modifiable factors, especially the correct education of future health care workers.

## Introduction

### Aging of society and the still existing problem of ageism

Due to the aging of societies in highly developed countries, there is an increasing global social burden with diseases of old age. In order to prevent these tendencies, special attention should be paid to prophylaxis, in the form of appropriate physical activity, adherence to proper dietary principles or maintaining proper mental activity (1). Adequate education of future medics in the correct principles of preventing premature occurrence of diseases of old age and proper management in the event of their occurrence will also play an important role, which may provide a greater number of necessary specialists.

While the very fact that investment in the education of future specialists in the treatment of old age diseases is necessary, the implementation itself is associated with many limitations, especially in the realities of highly developed societies. An example can be the situation described in Taiwan, where the society is aging so quickly that general social measures have been taken to educate not only younger people who will work in learned professions in the future, but also middle-aged and even advanced people (2). It is a favorable phenomenon from the point of view of involving seniors in social and scientific activity, but it shows a significant problem of the shortage of young people in which investing would lead to the creation of specialists who during their professional career help seniors.

The fact that it is worth investing in activities aimed at proper conduct toward the elderly population is evidenced by the experience of the Japanese society, which is characterized by rapid aging. The proportion of elderly people in Japan has quadrupled in the past 50 years, from 5.7% in 1960 to 23.1% in 2010, the fastest growth in the world. In order to deal with such a situation, ageism was fought, i.e., the phenomenon of exclusion on the basis of age. While creating networks of connections aimed at creating appropriate conditions for seniors to implement pro-health behaviors, thus reducing the frequency and speed of occurrence of negative effects of pathological aging, thus reducing the social costs related to the possible need for treatment (3). In such a model, which is a role model for other developed countries, the correct education of medical staff plays an important role.

In order for similar solutions to be introduced, the first step should be to limit the phenomenon of ageism in a given society. As proven in many studies, the exclusion of elderly people reduces cognitive functioning in seniors affected by this exclusion, mainly through the self-stereotyping mechanism, in which people in advanced age, who are discriminated against, begin to believe in stereotypes about themselves and thus limit their physical activity and mental (4). Until actions are taken to reduce the negative phenomenon of ageism and its consequences, attempts to create more advanced programs and actions aimed at activating seniors in order to prevent premature occurrence of diseases of old age, including education of future gerontologists, may not bring the expected results.

However, are there any methods to reduce the phenomenon of ageism, especially among young people who will be working with elderly people in the future? According to research conducted in Turkey on students of last years of nursing, there is a significant correlation between professional values, defined as individual standards effective in solving the problem of interaction with patients and making therapeutic decisions, and their correct attitudes toward people in advanced age (5). In this way, the very emphasis on developing the right models of behavior in students reduces the risk of the phenomenon of ageism in the medical community.

Another factor important in shaping attitudes that protect against ageism is the level of empathy. In a study of nursing students in China, three levels of empathy were identified: high (*n* = 42), medium (*n* = 399) and low (*n* = 181). During the analysis of the attitudes of individual groups toward elderly patients, it was noticed that the low level of empathy had a moderating role in the relationship between knowledge about aging and negative attitudes toward seniors (6). This means that even properly communicated knowledge about aging may not be able to reduce the phenomenon of ageism, if appropriate measures are not taken to increase empathy in future doctors.

In addition to the described dependence, one should not forget how important it is to promote correct attitudes from an early age. According to the analysis conducted by Teater and Chonody (7) among younger people entering the age of adolescence (11–13 years of age), contact with people at an advanced age in childhood was important in shaping positive attitudes toward seniors. Promoting a wide-ranging intergenerational integration already in childhood may very likely reduce the occurrence of the phenomenon of ageism in the future environment of medical personnel.

### Interventions in education on how to deal with the elderly

In order to shape appropriate attitudes of health care workers toward people of advanced age, taking into account the factors described above, it is necessary to ensure an appropriate level of education at the stages before starting work. According to a review by Hovey et al. such education produces the best results when it is introduced directly in a clinical setting and can be checked immediately. However, attention was drawn to the fact that the available literature has gaps, and the results of the analyzes should be confirmed in multicentre and large-scale studies (8). In turn, in another systematic review and meta-analysis, which analyzed 63 studies from 1976 to 2018, it was shown that the education of correct attitudes toward people in advanced age brings the best results when combined with active intergenerational integration (9), which is somehow an explanation of the effectiveness of education conducted in a clinical setting.

Noting the importance of intergenerational integration in the education of future doctors, one can refer to the study by Cadieux et al., which showed a correlation between the mentioned integration and stereotypes in predicting attitudes toward people in advanced age. It was also noted that a very important element of education is to refute stereotypes of incompetence, assuming that seniors are unable to perform certain advanced activities (10).

### The attitude of medics to the elderly and the effectiveness of treatment

Ageism undoubtedly affects the quality of doctor-patient communication and relationship. The way that healthcare practitioners communicate with eldery patients may impact treatment effectiveness and adherence. First of all, older people do not want to be called “elderly”. Medical providers should address older adults by their preferred terminology to ensure compliance (11).

Among nurses caring for older adults, perception of using cognitive behavior therapy might target ageism and is crucial for optimal clinical practice (12).

A very important aspect of elderly is coping with pain. Attitudes and beliefs of clinicians play an essential role in the care of older patients in pain. Ageism and the special situation of older people should be taken into account when talking about therapeutic choices and activity recommendations by health care professionals (13). On the other hand, patients' beliefs are also very important. One of the reasons why older adults not seek care for restricting back pain were beliefs about the age-related inevitability of restricting back pain (14).

Another important aspect is “age” in oncological treatment. Habr (15) claims that we must end ageism in cancer clinical trials. Older patients suffering from cancer are often excluded from clinical trials and are undertreated when compared to younger ones (16). One of the factors standing in the way of older people obtaining high-quality cancer treatment is the lack of clinical trial evidence that can help oncologists make informed decisions about the best possible treatment options. The data shows that older patients are underrepresented in oncological clinical trial research, people over 65 years old living with cancer represent only about 40 percent of enrollees in registration trials of new cancer therapies (15).

Increasing age was associated with markedly decreased rates of histological verification, surgery and chemotherapy in Scottish colorectal cancer patients. It is not possible to be sure whether there was ageism in the management of older patients with cancer, nevertheless, the rate of histological verification fell markedly with increasing age, making it questionable whether decisions to treat were based on best clinical practice at the time (17).

The negative consequences of ageism have an impact on the attitudes of those who deal with these people such as health care professionals, they are not limited to elderly people themselves. In everyday geriatric oncology practice, one should bear in mind the risk of stating that the patient is physiologically too old for a particular treatment (16).

### Purpose

The aim of the work was to answer research questions:

- What are the attitudes toward the elderly among students of medical and health sciences?
- Can attitudes toward the elderly among students of medical and health sciences depend on socio-geographic factors, field of study and seniority?
- Can the attitudes toward the elderly among students of medical and health sciences depend on private contacts with the elderly?

## Participants and methods

### Participants and data collection

A cross-sectional online survey was conducted between February and September 2021. Eight hundred and three medical and health sciences students participated in the research (616 women, 180 men, 28 respondents do not specify their gender) from various fields of medical studies (physiotherapy, nursing, medicine, psychology, pharmacy, medical analysis, dietetics, occupational therapy, audiophonology, laboratory diagnosis, obstetrics, electro-radiology, emergency medical and other).

Participation in the study was anonymous. Sampling was based on volunteers. We used an online platform (Survgo system), where an online questionnaire was placed and then posted on social media. Only students of medicine and healthcare facilities participated in the study. Before starting to complete the questionnaires, participants were informed that their responses were anonymous. Only fully completed questionnaires were included in the analysis.

### Procedure and measurements

#### Measurements

The first questionnaire contains socio-demographic questions about the represented ages, gender, place of residence, field of study and year of study.

The KAOP questionnaire consists of 34 items that measure attitudes toward older adults (see Supplementary Attachment 1). Seventeen of the items are negatively worded and 17 are positively worded (18). The scale was constructed using a 6-point summed Likert response attitude measure ranging from: 1 (strongly disagree), 2 (slightly disagree), 3 (disagree), 4 (agree), 5 (slightly agree), and 6 (strongly agree). To obtain the total score, scores for negatively worded items are reversed and added in with the positive responses. Scores for the KAOP range from 34 to 240 with higher scores represented a more positive attitude toward older adults (18, 19). Cronbach's α for the results of the KAOP questionnaire was estimated at the level of 0.86 with 95% confidence boundaries [0.85, 0.87] which corresponded to a good level of reliability (20).

The survey “Contact with older people” originally consisted of five single-choice questions. The characteristics of the survey are shown in Table 1.

**Table 1:** Characteristics of the “contact with older people” survey.

**Question**	**Response options**
Have you ever lived with an elderly person?	Yes
	No
Do you have contact with elderly people in your private life?	Never
	Only on occasion
	Occasionally
	Yes, few times a week
	Yes, I live/d with an elderly
Do you have contact with people over 65 at your college?	Yes
	No
	I have no occasion
Do you keep in touch with elderly people, who are not your family?	Yes
	No
	I have no occasion
How important are contacts with elderly people for you?	Not important
	Indifferent
	Important
	Very important

#### Ethical approval

The study was approved by the Bioethics Committee of the Nicolaus Copernicus University Collegium Medicum in Bydgoszcz, Poland (KB 83/2021). The research was conducted in accordance with the Helsinki Declaration. The survey was voluntary and anonymous.

#### Statistical analysis

Analyses were conducted using the R Statistical language (version 4.1.1; R Core Team, Vienna, Austria, 2021) on Windows 10 PRO 64 bit (build 19044), using the packages base (21), sjPlot [version 2.8.9; (22)], report (version 0.5.1; (23)), gstatsplot [version 0.9.0; (24)], and psych [version 2.1.6; (25)].

The significance level of the statistical tests in this analysis was considered to be α = 0.05.

The normality of the distributions of the variables was examined using the Shapiro-Wilk test and based on the calculated skewness and kurtosis parameters.

In the case of two groups Welch's *t*-test (with the assumption of inequality of variance) was used along with Hedges' effect size estimate, ĝ_*Hedges*_. The effect interpretations were based on Cohen's convention (26).

In the case of more than two groups the ANOVA Welch test was conducted with the partial omega-squared effect size,${\hat{\;w}}_{p}^{2}$. The effect size interpretations were based on Field's convention (27). For all-pairs comparisons in one-factorial layout with normally distributed residuals but unequal between-groups variances the Games-Howell test was performed.

The correlation test statistic was based on Pearson's product moment correlation coefficient and follows a *t* distribution with degrees of freedom length (*n*_*pairs*_ −2). An asymptotic confidence interval was given based on Fisher's Z transform.

A graphical representation of the comparisons is presented using a combination of box and violin plots along with separating data points in the between-subject designs, with detailed statistics attached to the plot as a subtitle.

The significance of the difference between the frequencies of the values within a variable was tested using the Pearson goodness-of-fit test^1^.

## Results

### Characteristics of the sample

Eight hundred and three medical and health sciences students participated in the study. The frequencies and shares of the sample by sex, age, place of residence, marital status, field of study and stage of study are shown in Table 2.

**Table 2:** Characteristics of the sample.

**Parameter**	**Group**	***N***	***n***	**Shares**	** $p_{\text{gof}}^{\text{a}}$ **
Gender	Female	796	616	77.3%	**<0.001**
	Male		180	22.7%	
Age	Up to 20 yrs.	803	225	28.0%	**<0.001**
	21–25 yrs.		236	29.4%	
	26–30 yrs.		12	1.4%	
	Over 30 yrs.		6	0.7%	
	Lack of data		324	40.5%	
Residence	Village	796	212	26.6%	**<0.001**
	Town <50 k		133	16.7%	
	City 50–100 k		65	8.2%	
	City 100–250 k		88	11.1%	
	City >250 k		298	37.4%	
Marital status	Single	796	306	38.4%	**<0.001**
	Informal relationship		219	27.5%	
	Married		253	31.8%	
	Other (separated, divorced, widower)		18	2.3%	
Major	Physiotherapy	789	453	57.4%	
	Nursing		35	4.4%	
	Medicine		101	12.8%	
	Psychology		46	5.8%	
	Pharmacy		25	3.2%	
	Medical analysis		24	3.0%	
	Dietetics		21	2.7%	
	Obstetrics		21	2.7%	
	Other^*^		63	8.0%	
Stage of study	BSc/ MSc, 1–3 yr.	619	568	91.8%	**<0.001**
	MSc. 4–5 yr.		43	6.9%	
	Supp. MSc.		8	1.3%	

* Occupational therapy, audiophonology, laboratory diagnosis, electroradiology, emergency medical.

a Pearson goodness-of-fit test.

Bold value indicates statistically significant values.

### Exploration the attitudes toward elderly (KAOP questionnaire) among medical and health sciences students

The descriptive statistics of the KAOP scores for the whole sample shown in Table 3. From the data in Table 3, it is shown that the distribution of KAOP scores meets the assumptions of a normal distribution (*p*_*Shapiro*−*Wilk*_ > *0.05)*.

**Table 3:** The descriptive statistics of KAOP scores with the normality test results, *N* = 803.

**M**	**SD**	**Mdn**	**IQR**	**Min**	**Max**.	**Sk**.	**Kurt**.	**W**	***p*_Shapiro − Wilk_**
131.8	17.1	131.0	23.0	74.0	177.0	−0.1	0.00	1.0	0.103

Bold value indicates statistically significant values.

#### The KAOP scores grouped by gender

Overall, women had lower attitudes toward elderly compared to men. The results of the KAOP questionnaire by gender are shown in Table 4.

**Table 4:** The KAOP scores grouped by gender, *N* = 796.

**Gender**	**KAOP scores^a^**	***p*_tWelch_**
Female	130.8 (16.9)	**0.002**
Male	135.3 (17.8)	

a In the form of M (SD)—here and hereafter.

Bold value indicates statistically significant values.

#### The KAOP scores grouped by age

Above average scores were obtained by students in the age groups of 21–25 yrs. and over 30 yrs. Below average scores were recorded for students up to 20 yrs. and 26–30 yrs (see Table 5).

**Table 5:** The KAOP scores grouped by age group, *N* = 803.

**Age group**	**KAOP scores**	***p*_F − Welch_**
Up to 20 yrs.	129.5 (18.0)	0.423
20–25 yrs.	131.6 (16.8)	
26–30 yrs.	127.4 (21.4)	
Over 30 yrs.	135.0 (20.9)	

#### The KAOP scores grouped by major factor

Scores on the KAOP questionnaire relative to the major ranged from 122.4 to 134.57. The highest scores were shown for physiotherapy students and the lowest for pharmacy students (see Table 6).

**Table 6:** The KAOP scores grouped by major (descending), *N* = 789.

**Major**	**KAOP scores**	***p*_F − Welch_**
Physiotherapy	134.6 (16.2)	**<0.001**
Psychology	133.6 (16.5)	
Dietetics	131.3 (16.9)	
Medical analysis	130.3 (15.6)	
Obstetrics	128.5 (13.0)	
Medicine	127.9 (19.1)	
Other	126.2 (17.4)	
Nursing	125.3 (15.3)	
Pharmacy	122.4 (19.8)	

#### Results of the KAOP questionnaire by study stage

The highest level of attitude Toward elderly was reported in students of 1st to 3rd year of study. For 4th-5th year or supplementary level students, attitude levels were decreasing (see Table 7).

**Table 7:** The KAOP scores grouped by age study stage, *N* = 619.

**Study stage**	**KAOP scores**	***p*_F − Welch_**
BSc/MSc 1–3 yr.	130.9 (17.2)	0.095
MSc 4–5 yr.	128.9 (18.5)	
Suppl. MSc	121.6 (11.3)	

### Exploring the relationships of attitudes toward elderly (KAOP questionnaire) among college students

#### The effect of the gender, on attitudes toward elderly

The Welch's *t*-test conducted showed that the level of attitude toward old people in men (*M* = 135.33, *SD* = 17.75, *n* = 180) was significantly higher than in women (*M* = 130.77, *SD* = 16.85, *n* = 616) with a small effect size, *t*_Welch_ (280.01) = −3.06, *p* = 0.002, ĝ_Hedges_ = −0.26.

#### The effect of the age factor on attitudes toward elderly

There was no significant correlation between the age factor on the quotient scale and the KOAP score, *r* = 0.03, *p* = 0.440, *df* = 597, *CI*_95%_ [−0.05, 0.11].

#### The effect of residence factor on attitudes toward elderly

**Figure 1:**
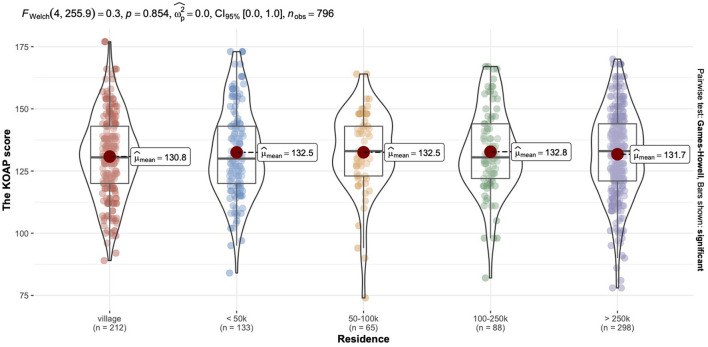
Comparative characteristics of the KAOP mean scores grouped by residence.

The range of mean scores by residence was [130.82, 132.85]. The ANOVA Welch analysis showed no significant differences between the residence factor and KAOP score (see Figure 1).

#### The effect of the of the marital status on attitudes toward elderly

**Figure 2:**
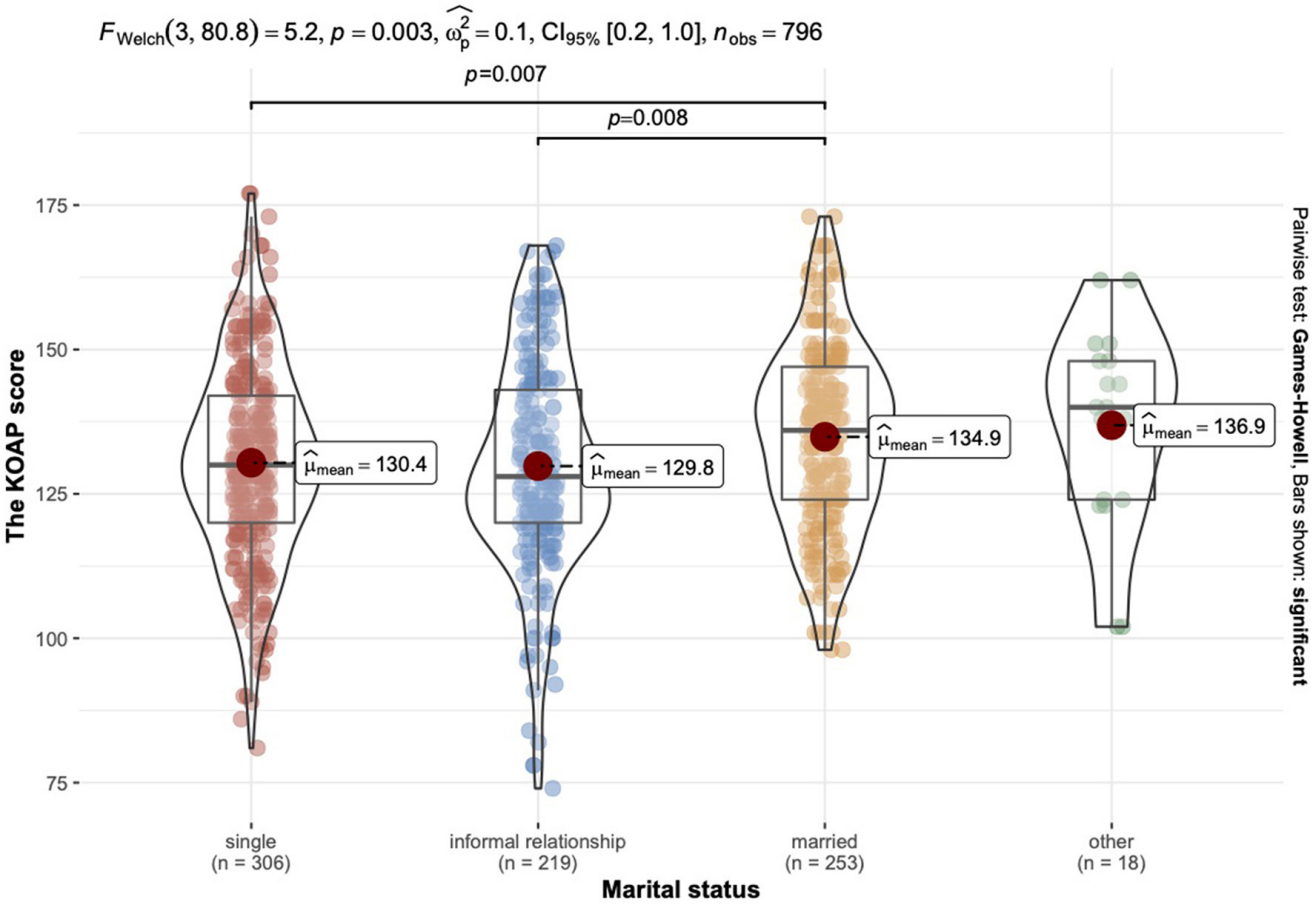
Comparative characteristics of the KAOP mean scores grouped by marital status.

Welch's ANOVA analysis showed that married individuals had significantly higher KAOP scores (*M* = 134.88, *SD* = 15.78) compared to singles (*M* = 130.82, *SD* = 17.06) and those in an informal relationship (*M* = 129.80, *SD* = 18.28) (see Figure 2).

#### The effect the of the major on attitudes toward elderly

**Figure 3:**
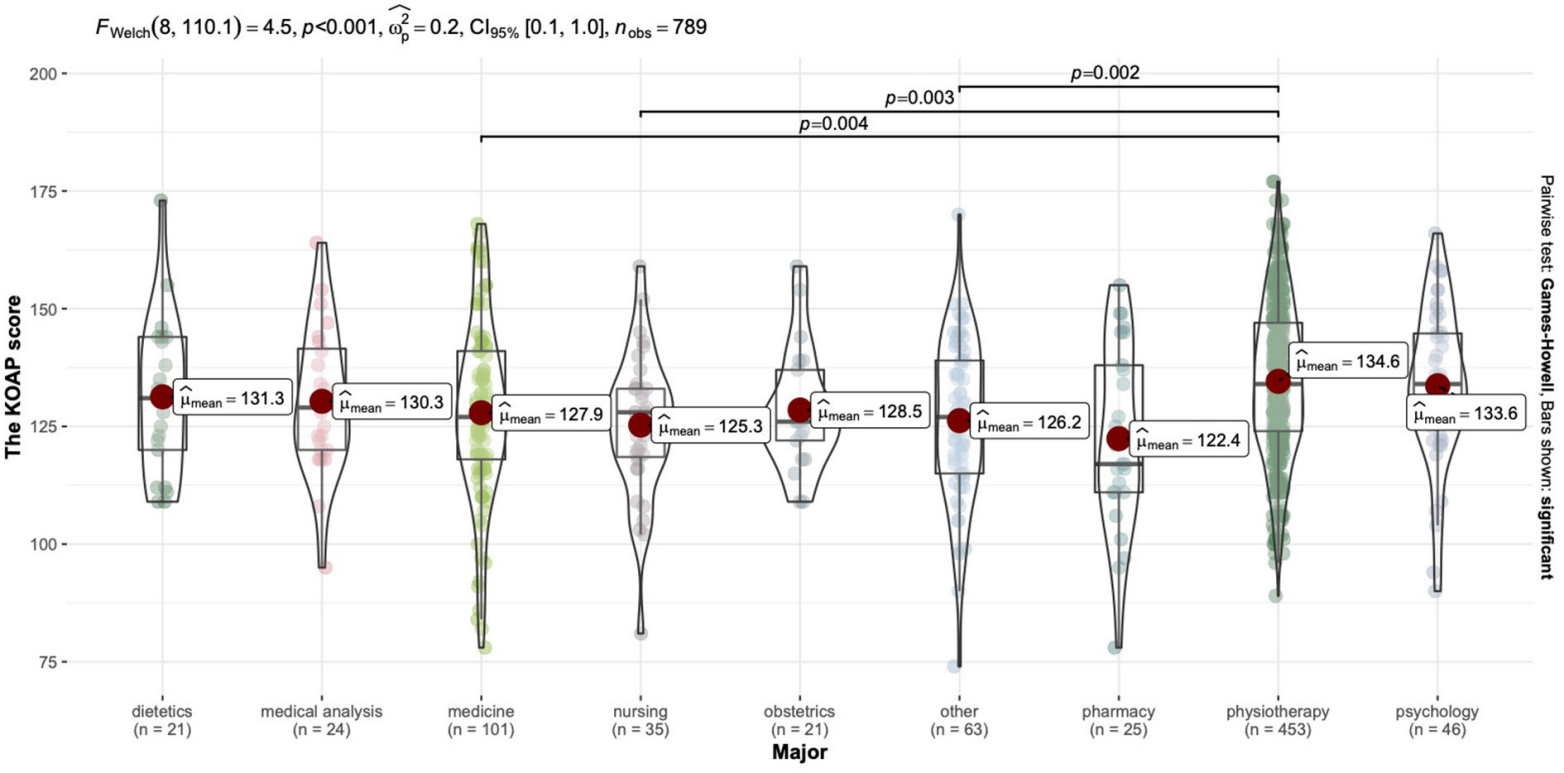
Comparative characteristics of the KAOP mean scores grouped by major.

Among the nine majors, the highest scores of attitudes toward elderly were explored in physiotherapy students (*M* = 134.57, *SD* = 16.17) and the lowest scores were observed in pharmacy students (*M* = 122.40, *SD* = 19.78). The KAOP scores of physiotherapy students were significantly higher the scores of medical, nursing students and other medical majors (see Figure 3).

#### The effect the study stage factor on attitudes toward elderly

**Figure 4:**
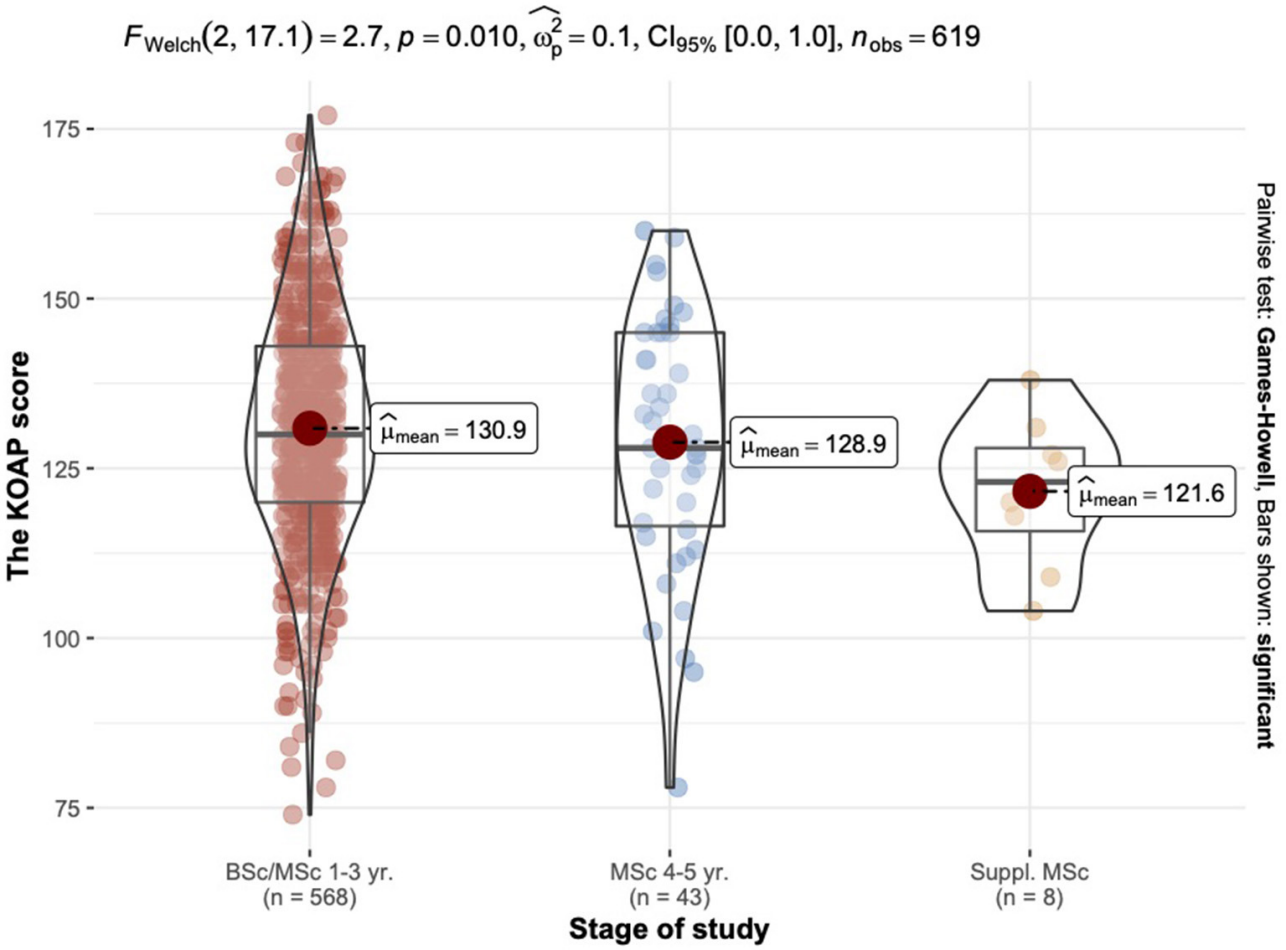
Comparative characteristics of the KAOP mean scores grouped by study stage.

The range of mean scores by residence was [121.62, 130.91]. The ANOVA Welch analysis showed no significant differences between the study stage factor and KAOP score (see Figure 4).

### Exploring the relationships of attitudes toward elderly (contact with older people survey) among college students

The survey questions along with the response rates are shown in Table 8. In addition, a separate column contains the average KAOP questionnaire results for each response option.

**Table 8:** Survey results with the KAOP questionnaire scores.

**Response option**	***n***	**Share**	**KAOP score**	***p*_F − Welch_**
**Have you ever lived with an elderly person? (*****N*** **=** **802)**
Yes	281	35.0%	130.1 (16.7)	0.041^*^
No	521	65.0%	132.7 (17.3)	
**Do you have contact with elderly people in your private life? (*****N*** **=** **802)**
Never	2	0.2%	134.5 (10.6)	0.058
Only on occasion	84	10.5%	132.8 (21.6)	
Occasionally	112	14.0%	127.5 (19.4)	
Yes, few times a week	503	62.7%	131.9 (16.1)	
Yes, I live/d with an elderly	101	12.6%	134.7 (14.5)	
**Do you have contact with people over 65 at your college? (*****N*** **=** **798)**
Yes	615	77.1%	132.5 (16.3)	0.145
No	105	13.2%	129.2 (18.8)	
I have no occasion	78	9.7%	129.3 (21.0)	
**Do you keep in touch with elderly people. who are not your family? (*****N*** **=** **802)**
Yes	416	51.9%	133.4 (15.5)	**0.013**
No	200	24.9%	128.7 (20.0)	
I have no occassion	186	23.2%	131.4 (16.9)	
**How important are contacts with elderly people for you? (*****N*** **=** **802)**
Not important	9	1.1%	108.4 (22.1)	**<0.001**
Indifferent	154	19.2%	128.5 (20.0)	
Important	433	54.0%	132.4 (16.6)	
Very important	206	25.7%	133.9 (14.6)	

* p_*tWelch*_. Bold value indicates statistically significant values.

From the data of Table 8, it is shown that almost 2/3 of the respondents co-habitated with the elderly. About 75% of people have contact with the elderly at least once a week in their private life, 77% of people have contact with the elderly at university. More than half of people have contact with the elderly without being in a family relationship with them. Nearly 80% of people indicated the importance of contact with the elderly.

Those who live and/or have regular contact with the elderly were characterized by higher KAOP questionnaire scores.

## Discussion

In an aging society, we need medical education to focus more on activities aimed at displaying positive attitudes toward older people. Results of presented research run among students from various fields of medical faculties, such as physiotherapy, nursing, medicine, psychology, pharmacy, medical analysis, dietetics, occupational therapy, audiophonology, laboratory diagnosis, obstetrics, electro-radiology and emergency medical confirm that need. Among the nine majors, the highest scores of attitude toward elderly were explored in physiotherapy students and the lowest scores were observed in pharmacy students. The KAOP scores of physiotherapy students were significantly higher than the scores of medical, nursing students and other medical majors. According to attitudes toward older adults, above average scores were obtained by students in the age groups of 21–25 yrs. and over 30 yrs. Below average scores were recorded for students up to 20 yrs. and 26–30 yrs. In the systematic review, where 63 studies were analyzed, ageism interventions demonstrated a strongly significant effect on attitudes, knowledge, and comfort. What is more, combined interventions with education and intergenerational contact showed the largest effects on attitudes. Stronger effects for females and for adolescent and young adult groups were found (9), while in our research the level of attitude toward old people in men was significantly higher than in women and there was no significant correlation between the age. A more positive attitude toward the elderly among women was also observed by Luo et al. (28). They assessed ageism among college students in the United States and China. However, it should be emphasized that medical students were not included in the study. Samra et al. (29), in their systematic review analyzed attitudes toward the elderly among medical students and doctors. The authors indicate that four of the five high-quality studies included in this review reported more positive attitudes in females than males.

What is important is that nearly 80% of students indicated the importance of contact with the elderly. Students who live and/or have regular contact with the elderly were characterized by higher KAOP questionnaire scores. The importance of intergenerational integration in the education of future doctors, was underlined in the study which showed a correlation between the mentioned integration and stereotypes in predicting attitudes toward people in advanced age (10). The study's results by Lee et al. (30) also indicate the importance of time spent with the elderly in eliminating the phenomenon of ageism. Among the doctors participating in the survey, ageism was negatively correlated with the percentage of elderly patients treated.

Zhao et al. (31), who assessed ageism among medical students in China, emphasizes that one of the factors increasing negative attitudes toward the elderly is the lack of caring experiences with older adults during clinical practice. This can be a barrier to choosing elderly care as a professional specialization pathway. As emphasized by Liu et al. (32), a very low proportion of the nursing students ranked caring for older people as their first choice of work.

The results of the cited studies indicate the need to expand the curricula with practical activities in direct contact with the elderly.

### Limitations

The study's main limitation was that although the invitation to fill in the survey was distributed in the same way in all faculties, the vast majority of participants were physiotherapy students (54.7%). The obtained percentage distribution of representatives of particular fields of study results from how the students responded to this invitation. All questionnaires were completed online. The study was carried out during the COVID-19 pandemic.

## Conclusion

The attitude toward the elderly depends on many factors, such as the chosen field of study, stage of education, contact with the elderly, gender or marital status. In order to develop proper contact with seniors, the solution may be to influence modifiable factors, especially the correct education of future health care workers. Well-trained medical staff is one of health care systems' most critical challenges worldwide. Considering the forecasts of the growing number of older adults and the phenomenon of ageism, educating well-prepared medical staff: nurses, physiotherapists, occupational therapists, and others seems to be a massive challenge for centers educating medical staff. Research in this area allows for a closer look at the phenomenon of ageism and many factors increasing or decreasing the intensity.

## Data availability statement

The raw data supporting the conclusions of this article will be made available by the authors, without undue reservation.

## Ethics statement

The study was approved by the Bioethics Committee of the Nicolaus Copernicus University Collegium Medicum in Bydgoszcz, Poland (KB 83/2021). Written informed consent for participation was not required for this study in accordance with the national legislation and the institutional requirements.

## Author contributions

Conceptualization: MP and KK-K. Methodology, formal analysis, investigation, writing—review and editing, visualization, and project administration: MP. Data curation and writing—original draft preparation: MP, AW, JH, and AP. Supervision: KK-K. All authors have read and agreed to the published version of the manuscript.

## Conflict of interest

The authors declare that the research was conducted in the absence of any commercial or financial relationships that could be construed as a potential conflict of interest.

## Publisher's note

All claims expressed in this article are solely those of the authors and do not necessarily represent those of their affiliated organizations, or those of the publisher, the editors and the reviewers. Any product that may be evaluated in this article, or claim that may be made by its manufacturer, is not guaranteed or endorsed by the publisher.

## Footnotes

^1^The null hypothesis assumed equal proportions between the categories of the variable.
