# Supplementary material for: Who will treat older patients? Should medical education focus more on activities aimed at displaying positive attitudes toward older people? The prevalence of ageism among students of medical and health sciences
Source: Front Public Health. 2022 Dec 1;10:1032487. doi: 10.3389/fpubh.2022.1032487 (PMC9752869; doi:10.3389/fpubh.2022.1032487)
Supplement: Supplementary file 1 [file Table_1.DOCX]

Attachment 1

**Characteristics of the KAOP questionnaire**

| *Nr* | *Wording* | *Question* |
| --- | --- | --- |
| 1 | Negative | It would probably be better if most old people lived in residential units with people their own age |
| 2 | Positive | It would probably be better if most people lived in residential units with younger people |
| 3 | Negative | There is something different about most people; it’s hard to find out what makes them tick |
| 4 | Positive | Most old people are really no different from anybody else; they’re as easy to understand as younger people |
| 5 | Negative | Most old people get set in their ways and are unable to change |
| 6 | Positive | Most old people are capable of new adjustments when the situation demands it |
| 7 | Negative | Most old people would prefer to quit work as soon as pensions or their children can support them |
| 8 | Positive | Most old people would prefer to continue working just as long as they possibly can rather than be dependent on anybody |
| 9 | Negative | Most old people tend to let their homes become shabby and unattractive |
| 10 | Positive | Most old people can generally be counted on to maintain a clean, attractive home |
| 11 | Negative | It is foolish to claim that wisdom comes with age |
| 12 | Positive | People grown wiser with the coming of old age |
| 13 | Negative | Old people have too much power in business and politics |
| 14 | Positive | Old people should have power in business and politics |
| 15 | Negative | Most old people make one feel ill at ease |
| 16 | Positive | Most old people are very relaxing to be with |
| 17 | Negative | Most old people bore others by their insistence on talking “about the good old days” |
| 18 | Positive | One of the most interesting and entertaining qualities of most old people is their accounts of their past experiences |
| 19 | Negative | Most old people spend too much time prying into the affairs of others and giving unsought advice |
| 20 | Positive | Most old people tend to keep to themselves and give advice only when asked |
| 21 | Negative | If old people expect to be liked, their first step is to try to get rid of their irritating faults |
| 22 | Positive | When you think about it, old people have the same faults as anybody else |
| 23 | Negative | In order to maintain a nice residential neighborhood, it would be best if too many old people did not live in it |
| 24 | Positive | You can count on finding a nice residential neighborhood when there is a sizeable number of old people living in it |
| 25 | Negative | There are a few exceptions, but in general most old people are pretty much alike |
| 26 | Positive | It is evident that most old people are very different from one another |
| 27 | Negative | Most old people should be more concerned with their personal appearance; they’re too untidy |
| 28 | Positive | Most old people seem quite clean and neat in their personal appearance |
| 29 | Negative | Most old people are irritable, grouchy, and unpleasant |
| 30 | Positive | Most old people are cheerful, agreeable, and good humored |
| 31 | Negative | Most old people are constantly complaining about the behavior of the younger generation |
| 32 | Positive | One seldom hears old people complaining about the behavior of the younger generation |
| 33 | Negative | Most old people make excessive demands for love and reassurance than anyone else |
| 34 | Positive | Most old people need no more love and reassurance than anyone else |
